# Practice and application of blended teaching based on RG-UClass in a preventive medicine course

**DOI:** 10.3389/fpubh.2026.1885005

**Published:** 2026-08-10

**Authors:** Sha Liu, Jiale Zhang, Yuqi Shang, Yufei Jiang, Xueyan Zhang, Zhiyong Hu, Caiyun Chen, Wei Mi

**Affiliations:** 1 School of Public Health, Shandong Medical and Pharmaceutical University, Yantai, China; 2 The First School of Clinical Medicine, Shandong Medical and Pharmaceutical University, Yantai, China; 3 The Second School of Clinical Medicine, Shandong Medical and Pharmaceutical University, Yantai, China

**Keywords:** blended teaching, course practice, preventive medicine, RG-UClass, teaching model

## Abstract

**Background:**

In the post-pandemic era, online teaching has become a cornerstone of higher education, especially for delivering structured medical knowledge. This study assessed the effectiveness of a blended teaching approach using the RG-UClass (Ruijie-UClass) smart platform in preventive medicine courses, aiming to provide empirical evidence of its impact and potential to improve learning outcomes.

**Methods:**

This quasi-experimental study enrolled 107 Grade 2019 clinical medicine students. Two intact classes were selected from all eight classes: Class 1 (*n* = 56, the control group) received traditional classroom teaching, while Class 2 (*n* = 51, the intervention group) received RG-UClass-based blended teaching. Course examinations and questionnaires were analyzed using the SPSS statistical software (Student’s *t*-test and *F*-test).

**Results:**

Students in the intervention group consistently outperformed the control group on pre-class, in-class, and post-course tests (*p* < 0.01), with the performance gap widening significantly over time (*F* = 23.615, *p* < 0.01). Furthermore, the intervention group obtained significantly higher total learning scores on the Student Engagement Scale (SES) compared with the control group (58.69 ± 11.34 vs. 50.96 ± 10.09; *t* = 3.997, *p* = 0.003), particularly in learning emotions, learning engagement, and learning performance (*p* < 0.0083). Notably, Biggs analysis also revealed significantly higher total deep learning scores (27.97 ± 6.24 vs. 25.04 ± 6.01; *t* = 3.981, *p* = 0.004) and deep learning strategy scores (14.85 ± 3.54 vs. 12.83 ± 2.89; *t* = 3.511, *p* = 0.006) in the intervention group.

**Conclusion:**

The RG-UClass-based blended model demonstrates reproducibility and adaptable flexibility, fostering student interest, initiative, and improved academic performance.

## 1 Introduction

The field of higher education in the 21st century has witnessed the rapid development of information technology and consequently, continuous innovation in educational concepts, which has resulted in online teaching gradually emerging as an important part of the education system. Especially in the post-pandemic era, the global education model has undergone unprecedented changes. At the beginning of the year 2022, the Ministry of Education in China put forward the requirements of “stopping classes without teaching” and “stopping classes without learning” and issued the “Guiding Opinions on the Organization and Management of Online Teaching in Higher Education Institutions during the infectious disease prevention and control period,” which required “ensuring that the quality of online learning is equivalent to that of offline classroom teaching” (1). This required using information technology to build a new learning model. In response to such requirements, intelligent education emerged as the optimal choice for achieving trans-formative development in the field of education (2, 3).

The traditional model of education was based on offline teaching and involved face-to-face lectures and the transmission of book knowledge and syllabic to students. The interaction between students and teachers was, therefore, limited to the classroom, and the teaching methods were limited by time and space (4, 5). In China, educators teaching preventive medicine courses often report that students are in a passive state of accepting knowledge during the learning process and lack initiative and creativity. In addition, clinical practice and critical thinking training are also lacking for these students. Different students exhibit different levels of knowledge, acceptance, and mastery (6), and it is difficult for teachers to keep track of each student’s learning progress, which leads to difficulty in organizing and managing learning tasks. Hence, exemplary teaching results are not easy to achieve. Especially in the face of the spread of infectious diseases, traditional instructional approaches have proven to be less effective than other teaching strategies in terms of practical application and pedagogical thinking ability. Blended teaching is a type of teaching that involves “online” + “offline” teaching mode, which integrates online teaching with traditional offline classrooms. This mode allows teachers to play a leading role in guiding, inspiring, and monitoring the teaching process while ensuring that students’ initiative, enthusiasm, and creativity are fully mobilized and reflected as the main body of learning (7). Online teaching is being used for various online courses in China, and in this type of teaching approach, students complete independent learning under the guidance of teachers. Offline classroom teaching, on the other hand, focuses on answering questions and solving problems, which strengthens the integration and higher-order application of knowledge (8). Therefore, the “online independent learning” + “offline flipped classroom” teaching model is an organic integration model that eliminates the time and space restrictions of the traditional education model to achieve complementary advantages, such as the mutual transition from passive learning to active learning and shift in knowledge acquisition from shallow to deep (9).

Smart education is an important direction in the field of educational reforms, which promotes education reform and development through a comprehensive integration of education, teaching, and information technology. The development of smart education adheres to the “classroom use, frequent use, and universal use” of information technology. Blended learning has been applied widely for teaching practice in many disciplines (10), such as neurological disease nursing, internal medicine, neurological nursing, and gynecology. A variety of blended teaching platforms are used commonly in medical teaching models, such as the Rainy Classroom, MOOC, SPOC, and BOPPPS teaching models (11–13). This report discusses a novel blended teaching model based on RG-UClass (14). RG-UClass is an interactive teaching system designed for university classroom instruction. As a multi-terminal, full-process integrated tool, it solves common classroom problems such as inadequate teacher-student communication, low student engagement, inefficient group discussion management, missing classroom activity records, and difficulty in tracking teaching effects. The system establishes a closed-loop teaching workflow that covers online pre-class learning and offline in-class interaction, with built-in functions like attendance tracking, real-time questioning, and case discussions, offering stronger interactivity than MOOC platforms. It integrates online and offline teaching tools into a single interface, captures whole-process teaching data, and supports teaching evaluation. These capabilities enable instructors to innovate blended teaching models, advance digital and intelligent teaching management, enhance teaching efficiency, and generate detailed data feedback for iterative teaching improvement. In China, RG-UClass has been used widely in the courses of many universities, such as Naval University of Engineering (15, 16).

The present study aimed to explore the practical application of RG-UClass and its impact on blended teaching and learning in the field of preventive medicine to provide a theoretical foundation and practical solutions for the promotion of wisdom education in university courses. The goal was to achieve improved learning interest and academic performance among students by optimizing the teaching model and enhancing the overall quality of blended teaching and students’ learning.

## 2 Materials and methods

### 2.1 Study subjects

This study adopted a quasi-experimental design. Preliminary questionnaires and statistical analyses were used to select two intact classes that were highly consistent in class size, male–female ratio, academic performance, and learning interest as research subjects. The participant recruitment period for this study was from September 1, 2024, to December 31, 2024. Eight Grade 2019 clinical medicine classes were initially screened. Based on their previous academic records, two classes with balanced baseline indicators were selected. A pre-survey covering all students in these two classes was then conducted for supplementary verification of baseline indicators. All study participants provided written informed consent for participation in this study. As the study included adult participants only, no parental or guardian consent was required. Among them, Class 1, the clinical medicine major of the class of 2019, formed the control group (*n* = 56), in which the traditional classroom teaching mode was used. Class 2, the clinical major of the class of 2019, formed the intervention group (*n* = 51), and the blended teaching mode based on RG-UClass was used for this group. This study recruited a total of 107 students from intact natural classes using convenience sampling. An *a priori* power analysis was not performed prior to data collection because the number of available intact classes was fixed and limited by the actual teaching arrangement.

### 2.2 Study methods

This study employed a quasi-experimental design. Two classes were selected from the eight clinical medicine classes of Grade 2019 cohort based on comprehensive matching criteria, including class size, student age, regular academic performance, and a pre-study learning interest assessment. Pre-intervention baseline data on academic mood, academic engagement, academic performance, and learning motivation were analyzed to assess group comparability. No statistically significant differences were found between the two groups (*p* > 0.05), as shown in Table 1.

**Table 1:** Baseline characteristics of research subjects (*x̄* ± *s*).

Baseline indicators	Control group (*n* = 56)	Intervention group (*n* = 51)	*t*	*p*
Age (years)	21.26 ± 1.03	21.08 ± 0.97	0.917	0.362
Last semester average GPA	78.35 ± 6.42	77.91 ± 6.15	0.366	0.714
Pre-study learning interest score	32.64 ± 4.18	32.17 ± 4.33	0.572	0.569
Pre-intervention learning motivation score	45.72 ± 5.36	44.98 ± 5.12	0.716	0.475

In this study, the two groups of students were subjected to different teaching approaches. The intervention involved using the RG-UClass model for educational activities. The control group students experienced teaching activities according to the traditional teaching model. Both cohorts completed the identical Preventive Medicine curriculum, which comprised 54 instructional hours (36 h of theoretical lectures and 18 h of laboratory practice). The two groups shared unified syllabi, matched homework loads, and identical textbooks; all instructional activities were delivered by a single instructor. Standardized observation protocols were applied consistently to all participants.

In the control group, prior to class, teachers prepared PPT slides in strict accordance with the unified syllabus. No online course framework, pre-class quizzes, or platform-based preview tasks were arranged. Students only completed simple textbook reading as basic preparation and were given no standardized online pre-learning assignments. All in-class instruction was delivered face-to-face in ordinary classrooms. All theoretical chapters (introduction, clinical preventive services, balanced nutrition, and chronic disease management) were taught exclusively offline, without any live teaching via smart platforms. Teachers projected the PPT slides manually during class. Each 80-min session consisted of a 40-min lecture by the teacher and a 40-min offline group discussion. Post-class, the teachers assigned paper-based homework of the same type and quantity as the intervention group. The homework was collected, graded offline, and returned to students with written feedback. Teachers then summarized classroom performance and adjusted the offline teaching plan for the next lesson.

**Figure 1:**
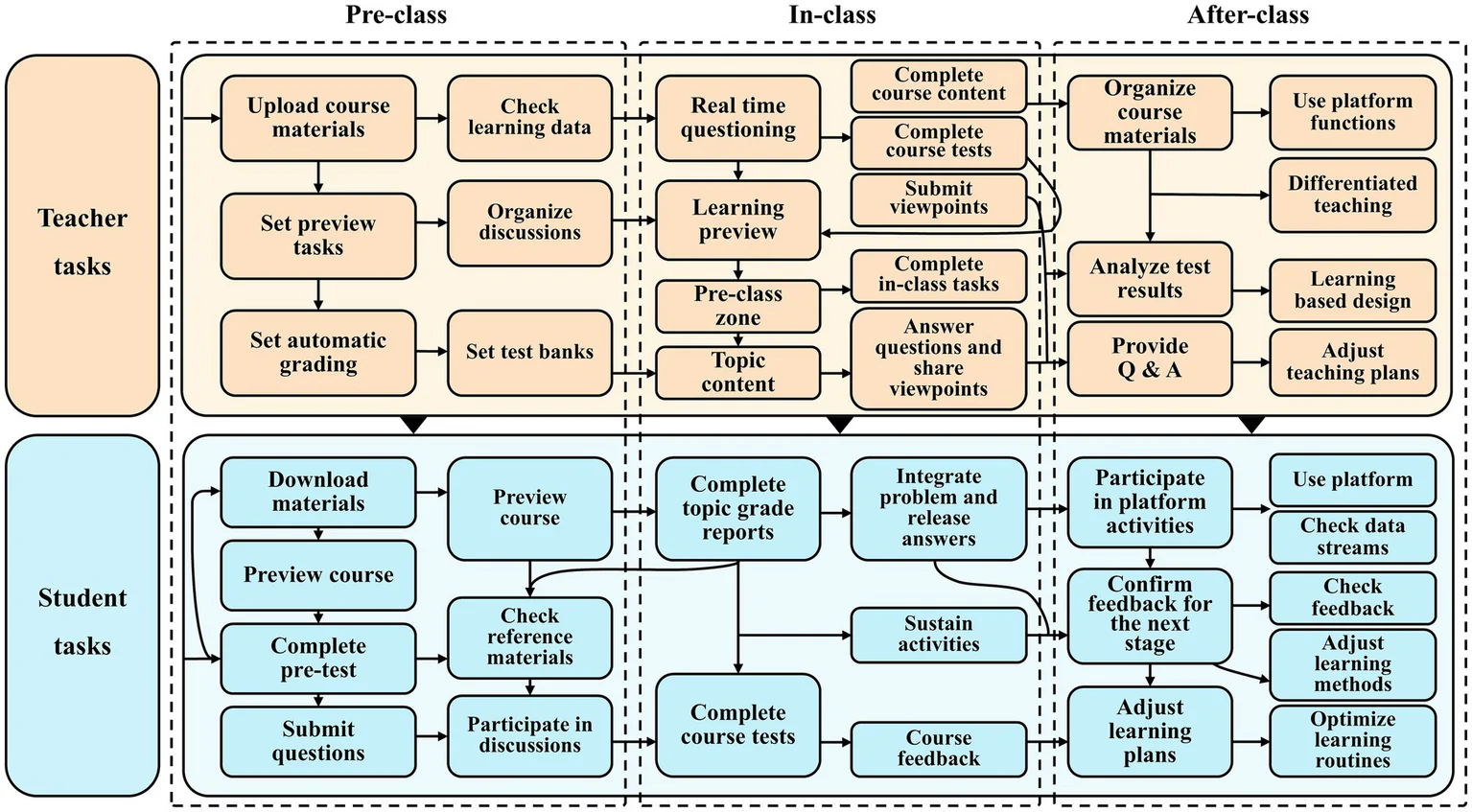
Flow chart of the RG-UClass-based hybrid teaching model.

In the intervention group, the RG-UClass model was used to carry out teaching through three stages. The flowchart of the RG-UClass blended teaching model is presented in Figure 1. Pre-class, the teacher set up the preventive medicine course on the RG-UClass intelligent platform and uploaded the preventive medicine chapter materials. Next, the teachers designed the “preventive medicine chapter content course tree” structure on the platform, which included a course chapter introduction, chapter learning objectives, pre-course discussion questions, and pre-course test questions. One week before the start of the course, students were notified to preview and test the platform. Second, online and offline teaching methods were selected according to the characteristics of the preventive medicine chapter content. For theoretical knowledge-based content, chapters on introduction, clinical preventive services, rational nutrition, chronic disease management, etc., RG-UClass has an intelligent platform for teaching online, which uses the lecture materials uploaded to the platform, and after clicking on the “teaching content,” opens the screen casting mode to live-stream the lecture at the designated time. The platform regularly uses attendance, new discussions, new Q&A, preemptive answers, and secondary preemptive answer annotations, along with other functions, and all the learning results and learning outcomes are retained in the platform. Offline classroom lecture mode is selected for practical knowledge-based content (cohort studies, case–control studies, screening experiments, etc.). This mode is used for topics that require students to understand through face-to-face interactive discussions, case studies, and demonstrations of methodological processes. Such an approach facilitates more intuitive understanding and intensive learning. Post-class, teachers can post assignments, submit assignments, and correct assignments on the RG-UClass Wisdom Platform. The platform automatically marks and scores the students’ homework results, and teachers can label excellent assignments as “typical,” while bad assignments can be marked and sent back for revision. After the lesson, the platform automatically pushes the lesson report, generates attendance records and interaction records, and continues the discussion. Additionally, the platform maintains a record of all discussions and board results for both teachers and students. This allows teachers to identify areas for improvement in their teaching, while students can review their performance.

The RG-UClass platform provides several core functional supports for this blended teaching model. Teachers can build a hierarchical course tree centered on knowledge points, integrating learning resources across all stages and dynamically adjusting content push as needed. The platform sets up independent discussion zones for the pre-class, in-class, and post-class phases, allowing teachers to pin commonly confusing questions, answer inquiries in batches, and export discussion data to identify class-wide learning difficulties. Its automatic grading module matches objective items against a standard answer library with an identification accuracy of over 98.7%, while subjective questions adopt intelligent keyword scoring, supplemented by a secondary manual review by teachers to calibrate score deviations and ensure reliable evaluation results.

### 2.3 Curriculum test

The curriculum test is an examination conducted to assess the learning situation of students after the implementation of the curriculum. Pre-class assessments, such as placement tests, are designed to gauge students’ prior knowledge and help teachers tailor their instruction accordingly. The first round of pre-class testing was administered on September 1, 2024, after students had completed all pre-class learning materials, in order to assess how well they had reviewed the pre-assigned knowledge. In-class tests, such as quizzes, serve as an immediate measure of students’ mastery of newly taught content. This assessment was conducted on November 5 to evaluate students’ learning performance since the start of formal instruction. The post-class test is a comprehensive assessment of students’ overall mastery of the course content. Upon course completion, a formative assessment (the post-class test) was implemented for both the control and intervention groups; this final test was administered on December 30, 1 week after the course ended. Students in both groups were given a stage test after pre-course knowledge, during-course knowledge, and after post-course knowledge review. Both groups of students were given the same test questions for the pre-course, in-class, and post-course tests. The three tests increased in difficulty sequentially, each lasting 30 min, and scores were based on a percentage system.

### 2.4 Questionnaire survey

#### 2.4.1 Curriculum engagement assessment questionnaire

The Student Engagement Scale (SES) (17) was adopted to assess students’ approach to learning methods, emotions, engagement, and performance. The scoring was as follows: 1 = not like me at all, 2 = not quite like me, 3 = not sure, 4 = like me, and 5 = very much like me. Forty students from the intervention group and forty students from the control group were randomly selected for the survey, and a total of 80 students were selected to complete the SES questionnaire for the pre-survey. The reliability and validity of the questionnaire were good, with a pre-survey Cronbach’s coefficient of 0.811 and adequate construct validity (KMO = 0.754, Bartlett’s test of sphericity *p* < 0.01). The results of the survey were consistent with the results of the formal experiment.

#### 2.4.2 Learning process questionnaire

The Biggs questionnaire (18) was adopted. It included items to assess students’ deep and shallow learning styles, each containing learning strategies and motivation. The scoring system was as follows: 1 = never, 2 = sometimes, 3 = half of each, 4 = often, and 5 = always. Forty students from the intervention group and forty from the control group were randomly selected for the survey, and a total of 80 students were selected to complete the Biggs questionnaire for the pre-survey. The reliability and validity of the questionnaire were good, with a pre-survey Cronbach’s *ɑ* of 0.826 and adequate construct validity (KMO = 0.714, Bartlett’s test of sphericity *p* < 0.01). The results of the survey were consistent with the results of the formal experiment. The results of the survey were consistent with the results of the formal experiment.

By combining the SES and Biggs’s Revised Two-Factor Study Process Questionnaire, this study captures two distinct dimensions of learning. The SES reflects students’ real-time learning engagement elicited by blended teaching, whereas the Biggs questionnaire reveals inherent differences in their learning approaches. Integrating these two instruments allows for a comprehensive analysis of how the RG-UClass-supported intervention affects students in terms of behavioral engagement and cognitive learning strategies.

### 2.5 Statistical analysis

All statistical analyses were performed using the SPSS 25.0 software. The measurement data were expressed as means ± standard deviations (*x̄* ± *s*). Group comparisons on each questionnaire dimension were conducted using independent *t*-tests, with the Bonferroni correction applied to adjust *p*-values for multiple comparisons. Moreover, the means of the three performance differences before, during, and after the class were compared using two-factor repeated-measures ANOVA with a test level of *ɑ* = 0.05.

## 3 Results

### 3.1 Baseline characteristics of research subjects

Table 1 summarizes the baseline indicators of the control group (*n* = 56) and the intervention group (*n* = 51), including student age, last semester academic performance (GPA), pre-study learning interest score, and pre-intervention learning motivation score. Independent-samples *t*-tests revealed no statistically significant differences between the two groups on any of these baseline indicators (all *p* > 0.05). These results confirm that the two groups were well matched at baseline, thereby eliminating the interference of confounding baseline factors in the subsequent comparison of teaching effectiveness.

### 3.2 Comparison of the three-stage test scores

Statistical analysis revealed that the intervention group consistently outperformed the control group on the pre-course, in-class, and post-course tests (*p* < 0.01). The differences in the test scores between the intervention and control groups gradually increased when moving from the pre-intervention to mid-intervention to post-intervention tests (*F =* 23.615, *p =* 0.001) (Mauchly’s test confirmed that the assumption of sphericity was met; therefore, unadjusted *F* values were reported), as shown in Tables 2, 3.

**Table 2:** Comparison of students’ performance between the control and intervention groups (*x̄* ± *s*) in the three stages of test results.

Groups	Pre-course test results	In-class test results	Post-course test results
Intervention group (*n* = 51)	83.62 ± 13.56	85.79 ± 13.78	89.77 ± 15.64
Control group (*n* = 56)	78.25 ± 11.34	78.21 ± 12.18	80.51 ± 12.81
*t*	3.390	4.782	5.247
*p*	0.001	0.000	0.000

**Table 3:** Three stages of concrete analysis (*x̄* ± *s*).

Groups	Pre-course test results	In-class test results	Post-course test results	Repeated measure analysis of variance		
				*F*	*p*	Biased *ȵ*^2^
Intervention group (*n* = 51)	83.62 ± 13.56	85.79 ± 13.78	89.77 ± 15.64			
Control group (*n* = 56)	78.25 ± 11.34	78.21 ± 12.18	80.51 ± 12.81			
Class main effect				23.615	0.000	0.50
Measure the number of main effects				50.91	0.000	0.70
Group number of tests				9.61	0.001	0.30

### 3.3 Results of the SES questionnaire

The SES questionnaire was completed by 56 students in the control group and 51 students in the intervention group. The analysis of the questionnaire data revealed significant differences between the two groups. Specifically, the total learning scores of the students in the intervention group was higher (58.69 ± 11.34) than that in the control group (50.96 ± 10.09) (*t =* 3.997, *p =* 0.003). The students in the intervention group presented high-performance scores in the three dimensions of learning emotions, learning engagement, and learning performance (*p* < 0.0083), as shown in Table 4 and Supplementary Table S1.

**Table 4:** Statistical results of SES questionnaires (*x̄* ± *s*).

Questionnaires	Items	Intervention group (*n* = 51)	Control group (*n* = 56)	*t*	*p*
SES	Learning methods	16.16 ± 2.89	14.72 ± 2.18	2.188	0.039
	Learning emotions	20.39 ± 3.23	18.48 ± 2.67	3.316	0.001
	Learning engagement	14.69 ± 2.04	11.90 ± 1.54	7.925	0.000
	Learning performance	7.45 ± 1.21	5.86 ± 1.67	5.675	0.000
	Total learning scores	58.69 ± 11.34	50.96 ± 10.09	3.997	0.003

### 3.4 Results of the Biggs questionnaire

The Biggs questionnaire was completed by 56 students in the control group and 51 students in the intervention group. The results revealed that the total score of deep learning in the intervention group (27.97 ± 6.24) was greater than that in the control group (25.04 ± 6.01) (*t =* 3.981, *p =* 0.004). The intervention group outperformed the control group in terms of deep learning strategies (*t =* 3.511, *p =* 0.006). However, no significant difference between the two groups was noted in terms of the total score of shallow learning (*t = −*1.854, *p =* 0.078). Notably, the intervention group scored lower in terms of shallow motivation to learn than the control group (*t = −*3.402, *p =* 0.007), as shown in Table 3. Further analysis revealed that in the deep learning motivation dimension, the intervention group scored higher than the control group in terms of seeking answers to questions in most classes (*t =* 4.001, *p =* 0.000). In addition, for the deep learning motivation dimension, the intervention group often took extra time to gain more knowledge (3.85 ± 1.41) and spent much of their time outside the classroom researching and discussing problems (3.78 ± 1.15) compared to the control group (*p* < 0.0031). In the shallow learning motivation dimension, the students in the intervention group who spent the least effort to complete the course (2.26 ± 1.14) scored lower (*t =* 4.679, *p =* 0.000), as shown in Table 5 and Supplementary Table S2.

**Table 5:** Statistical results of Biggs questionnaires (*x̄* ± *s*).

Questionnaires	Items	Intervention group (*n* = 51)	Control group (*n* = 56)	*t*	*p*
Biggs	Deep learning motivation	13.12 ± 3.01	13.21 ± 3.33	0.078	0.818
	Deep learning strategies	14.85 ± 3.54	12.83 ± 2.89	3.511	0.006
	Total deep learning scores	27.97 ± 6.24	25.04 ± 6.01	3.981	0.004
	Superficial motivation for learning	8.73 ± 2.31	10.52 ± 3.21	−3.402	0.007
	Shallow learning strategies	8.56 ± 2.47	9.13 ± 2.68	−1.599	0.097
	Total shallow learning scores	17.29 ± 5.55	19.65 ± 6.01	−1.854	0.078

## 4 Discussion

This study discusses the first application of the RG-UClass teaching model in the field of preventive medicine, and the results suggested that this model can significantly improve students’ learning ability and interest in learning. This efficacy was attributed to the optimized convenience and efficiency of this new teaching mode in the information technology domain. This teaching model enhances students’ motivation and initiative in learning and facilitates the “student, problem, and discussion-centered” innovative ability development. Therefore, the model can serve as a novel tool for educators to implement innovative teaching methods and for digital intelligent teaching management reform.

### 4.1 Course test performance analysis

As indicated by the findings of the course stage examinations conducted in this study, the implementation of the RG-UClass-based blended teaching method in the preventive medicine course yielded significant positive outcomes. The scores of the students who used this novel teaching model surpassed those of the students in the control group in the pre-course, in-class, and post-course tests. Furthermore, the disparity between the stage test scores demonstrated a consistent upward trend. Engaging in education using online learning platforms serves to fully harness the enthusiasm of students while ensuring that the independent thought process of each student is facilitated. Moreover, teachers ask guided thinking questions during the lesson, which can help students establish independent ways to solve problems and thereby gain a better understanding of the knowledge framework of preventive medicine and relevant cutting-edge research, which improves their learning performance gradually by enhancing their exploratory thinking. The online preview before class used in this model helped students establish the knowledge framework, and the combination of online and offline teaching in class enhanced the systematization and application of knowledge. The online discussion and homework feedback after class strengthened the learning results.

### 4.2 SES questionnaire results

In addition, the results of the SES questionnaire revealed that the implementation of the RG-UClass-based blended teaching method in preventive medicine courses resulted in good attitudes, participation, and performance among students in terms of learning. The significant total score difference between the two groups was not attributable to the learning methods dimension; instead, it was jointly driven by intergroup differences in learning participation and learning performance.

The total learning scores of the intervention group students who learned through the RG-UClass hybrid teaching platform were significantly different (*p* = 0.003) from those of the control group students. In addition, the two groups significantly differed in terms of the learning emotions (*t =* 3.316, *p =* 0.001), learning engagement (*t =* 7.925, *p =* 0.000), and learning performance (*t =* 5.675, *p =* 0.000). Before class, the students were guided to pre-study through online teaching; during class, online and offline lectures were combined with theoretical knowledge points for a systematic sorting-out and application; after class, the online teachers and students discussed key points and explored cutting-edge research knowledge. In regard to the application of the online teaching model through BOPPPS in the clinical internships of undergraduate dentistry students, the results of the SES questionnaire revealed differences in the total scores of students between the intervention group and the control group, although these differences were not statistically significant (*p* > 0.05). Strategies applied through some platforms in live teaching have been well described in the literature, which reports that learning efficiency can be improved with some creativity (19, 20). The difference in the total scores of the learning methods (*p* > 0.0083) observed between the two groups of students in this study was not statistically significant. Therefore, the results of the SES questionnaire suggested that the new mixed teaching mode based on RG-UClass allows for effective interactions between teachers and students, achieving outcomes similar to those reported by Jin et al. (21). Students’ active participation in class and initiative in the after-class discussions was significantly improved using this method, and this “student-centered” teaching model provided students with more independent learning opportunities and helped them cultivate critical thinking and problem-solving skills. Similar to the previous study (22), the combination of online and offline teaching methods greatly improved students’ learning interest and resulted in effective learning habits, which would continue to improve the academic performance of students.

### 4.3 Biggs questionnaire results

The Biggs questionnaire results indicated that students in the RG-UClass-based blended preventive medicine course tended to achieve higher total deep learning scores compared with the control group (*p* = 0.004). Specifically, the intervention group showed significantly higher scores only in the deep learning strategies dimension (*t* = 3.511, *p* = 0.006), whereas deep learning motivation exhibited no significant between-group difference. These findings demonstrate that the RG-UClass interactive teaching system primarily reshapes students’ cognitive learning strategies rather than altering their intrinsic learning motivation. Equipped with a rich array of interactive classroom modules, real-time feedback channels, and precise targeted knowledge analysis functions, the system continuously encourages students to adopt active, reflective, and well-structured learning behaviors. The higher overall deep learning scores observed in the intervention group stem mainly from improvements in deep learning strategies, rather than from any enhancement of students’ intrinsic deep learning motivation. It optimizes students’ learning behaviors and cognitive processing modes yet exerts little influence on their intrinsic willingness to engage in deep learning. Similar to the Mosleh et al.’s (23) study, the teacher’s teaching strategy improved in the intervention group in this study, and the new teaching model designed by the teacher was tightly interlinked, student-centered, focused on students’ learning experience, and could be promptly adapted to different teaching sessions. The results of the Biggs questionnaire indicated that through the implementation of the RG-UClass-based hybrid teaching model in preventive medicine courses, students’ participation could be improved, and teaching effectiveness could be enhanced. Therefore, the new model will play an important role in promoting the reform of practice teaching. Through the interactive function of the platform, students can actively explore knowledge, integrate information, and conduct in-depth discussions outside the classroom. This deep learning-based teaching method not only improves students’ academic performance but also lays a solid foundation for students’ future medical practice. Consistent with Xu et al. (24), who confirmed that the integrated BOPPPS and CBL teaching approach for clinical undergraduates in pathology experimental classes produced no significant between-group difference in the total shallow learning scale scores (*p* > 0.05), and that participants receiving the combined teaching attained markedly lower scores on the shallow learning strategy subdimensions (*p* < 0.01). Although the intervention group scored significantly lower on shallow learning motivation—indicating that students were less inclined toward perfunctory memorization and rote learning—mandatory learning tasks, such as basic knowledge memorization and fundamental exercises, remained required for all students. Consequently, no statistically significant difference was found in the total shallow learning scores between the two groups. This finding is in line with these studies (25–27), which state that the lack of an effective supervision mechanism for online teaching leads to students being easily distracted, and it becomes difficult for teachers to track and evaluate students’ learning status in real-time.

### 4.4 Limitations of this study

This study has certain limitations in terms of a short implementation period and scale. Unfortunately, there are few applications of the RG-UClass hybrid teaching model in teaching practice, and few lessons have to be learned. It is necessary to further expand the types of courses included in this research and application field, in addition to continuously improving the teaching content and updating the teaching resource library of the platform to provide a theoretical basis and practical solutions for the promotion of smart platforms and the implementation of hybrid teaching (28, 29). This quasi-experiment used a convenience sample of fixed intact classes and did not include an *a priori* power analysis. The sample of 107 students may have insufficient statistical power to detect small between-group differences. Moreover, the influence of confounding factors cannot be ruled out in this experiment. For example, students could have different levels of acceptance of new things, different levels of love for medical learning, and different experiences in terms of personal growth. The integrated mixed teaching model that combines online live lectures with offline flipped learning in the intervention group makes it impossible to separate the independent effect of any single teaching format, resulting in confounding effects. Future parallel controlled trials adopting various intelligent teaching platforms can be implemented to further disentangle the independent contributions of platform-specific functional modules and blended teaching philosophies to students’ learning performance. In addition, extended longitudinal follow-up studies spanning multiple semesters or academic years are warranted to capture the sustained long-term effects of the proposed teaching model.

## 5 Conclusion

This study aimed to investigate the impact of the RG-UClass intelligent teaching platform on student achievement. A quasi-experimental study was conducted, in which the SES and Biggs questionnaires were administered. The results indicated that the initial implementation of the RG-UClass teaching model in preventive medicine was statistically associated with improvements in students’ learning ability and learning interest, suggesting that this platform may have a positive effect on learning performance. The SES findings suggested that the blended teaching model was associated with more favorable learning attitudes, greater classroom engagement, and better academic performance. Participants in the intervention group demonstrated improved engagement and learning outcomes, as well as a greater willingness to participate in class discussions, which may foster the development of critical thinking and problem-solving skills. The Biggs questionnaire results further showed comparatively higher overall scores in deep learning and deep strategy among students who received the blended intervention. This pattern implies that these participants tended to engage in more active knowledge exploration and in-depth peer exchange, potentially building a solid foundation for their subsequent clinical practice. The RG-UClass platform enables a hybrid “online + offline” teaching model and a “student-centered, problem-centered, discussion-centered” approach. It effectively bridges gaps in classroom participation, teaching data recording, and result retention, and is therefore recommended for seamless integration of teaching practices.

## Data Availability

The original contributions presented in the study are included in the article/Supplementary material, further inquiries can be directed to the corresponding authors.
