# Supplementary material for: Practice and application of blended teaching based on RG-UClass in a preventive medicine course
Source: Front Public Health. 2026 Aug 10;14:1885005. doi: 10.3389/fpubh.2026.1885005 (PMC13500768; doi:10.3389/fpubh.2026.1885005)
Supplement: Supplementary file 1 [file Table_1.DOCX]

**Supplementary Material**

**Table S1.** Results of the SES questionnaire (*x̄* ± *s*)

| Questionnaires | Items | Intervention group (*n* = 51) | Control group (*n* = 56) | *t* | *p* |
| --- | --- | --- | --- | --- | --- |
| Learning methods | Pre-learning before class | 3.78 ± 0.81 | 3.21 ± 0.57 | 4.215 | 0.000 |
|  | Careful study in class | 4.11 ± 0.92 | 3.95 ± 0.71 | 0.987 | 0.325 |
|  | Review after class | 3.95 ± 0.75 | 3.42 ± 0.52 | 4.132 | 0.000 |
|  | Take good notes | 4.32 ± 0.82 | 4.14 ± 0.71 | 1.206 | 0.230 |
| Learning  emotions | Do your best | 4.21 ± 0.84 | 4.05 ± 0.82 | 0.993 | 0.322 |
|  | Theory and practice | 4.00 ± 0.75 | 3.54 ± 0.68 | 3.301 | 0.001 |
|  | Practical application of what you learn | 4.01 ± 0.75 | 3.60 ± 0.71 | 3.000 | 0.002 |
|  | Maintaining interest in course | 4.28 ± 0.85 | 3.62 ± 0.89 | 3.986 | 0.000 |
|  | Truly eager to learn | 3.89 ± 0.72 | 3.67 ± 0.68 | 1.999 | 0.234 |
| Learning engagement | Active group discussion | 3.92 ± 0.78 | 3.01 ± 0.76 | 5.211 | 0.000 |
|  | Participate in online chat | 3.41 ± 0.59 | 2.99 ± 1.11 | 2.431 | 0.028 |
|  | Frequent discussions and speeches | 3.61 ± 0.77 | 3.01 ± 0.68 | 4.307 | 0.000 |
|  | Help other students | 3.75 ± 0.69 | 2.89 ± 0.59 | 6.208 | 0.000 |
| Learning Performance | Good performance in the quiz | 3.89 ± 0.99 | 3.01 ± 0.67 | 5.954 | 0.000 |
|  | High scores | 3.56 ± 0.74 | 2.85 ± 0.67 | 5.193 | 0.000 |

**Table S2.** Results of the Biggs questionnaire (*x̄* ± *s*)

| Surveys | Items | Intervention group (*n* = 51) | Control group (*n* = 56) | *t* | *p* |
| --- | --- | --- | --- | --- | --- |
| Deep learning motivation | There is a deep sense of satisfaction in learning | 3.74 ± 1.21 | 3.58 ± 1.11 | 0.887 | 0.568 |
|  | Only diving into any problem is interesting | 3.77 ± 1.18 | 3.50 ± 1.07 | 0.974 | 0.498 |
|  | Studying hard because it is fun | 3.41 ± 0.95 | 3.01 ± 1.03 | 1.115 | 0.321 |
|  | Seek answers with questions in most classes | 3.12 ± 1.24 | 2.20 ± 0.81 | 4.001 | 0.000 |
| Deep learning strategies | A lot of work to get answers before you are satisfied | 3.61 ± 1.31 | 3.14 ± 0.10 | 3.002 | 0.003 |
|  | often take extra time to gain more knowledge | 3.85 ± 1.41 | 3.00 ± 1.10 | 4.211 | 0.000 |
|  | Continuous learning to understand key issues | 3.61 ± 1.25 | 3.85 ± 1.41 | 1.015 | 0.357 |
|  | Spend a lot of time outside of class researching and discussing issues | 3.78 ± 1.15 | 2.84 ± 1.01 | 4.898 | 0.000 |
| Superficial motivation for learning | Spend minimum effort to pass this course | 2.26 ± 1.14 | 3.58 ± 1.31 | 4.679 | 0.000 |
|  | Do as little as possible if you do not find the course interesting | 2.38 ± 1.08 | 2.48 ± 1.17 | 0.856 | 0.574 |
|  | Remember only the key points without trying to understand them | 2.01 ± 1.06 | 2.27 ± 1.31 | 1.874 | 0.167 |
|  | It is not necessary to read what is not tested | 2.08 ± 1.24 | 2.19 ± 0.99 | 1.564 | 0.224 |
| Shallow learning strategies | Seriously study only what is required for the course syllabus | 2.58 ± 1.25 | 2.74 ± 1.21 | 0.996 | 0.499 |
|  | rote memorization of points you do not understand | 2.68 ± 1.14 | 2.89 ± 1.05 | 0.917 | 0.501 |
|  | There is no need to do anything other than study | 1.64 ± 1.03 | 1.82 ± 1.03 | 0.885 | 0.641 |
|  | The best way to pass an exam is to memorize the test points | 1.66 ± 1.08 | 1.68 ± 1.11 | 0.548 | 0.987 |
